# Interruption of the circulation of an indigenous measles genotype and the introduction of other genotypes after a mass vaccination campaign in the Philippines

**DOI:** 10.1002/jmv.22103

**Published:** 2011-08

**Authors:** Naoko Fuji, Akira Suzuki, Mariko Saito, Rex Centeno, Hazel Galang, Socorro Lupisan, Remigio Olveda, Hitoshi Oshitan

**Affiliations:** 1 Department of Virology, Tohoku University Graduate School of MedicineSendai, Miyagi, Japan; 2 Tohoku-RITM Collaborating Research Center for Emerging and Reemerging Infectious DiseasesMuntinlupa, Philippines; 3 Research Institute for Tropical MedicineMuntinlupa, Philippines

**Keywords:** measles, molecular epidemiology, genotyping

## Abstract

Molecular analysis of measles viruses in the Philippines was conducted from 2000 to 2008. No confirmed measles cases were detected in the surveillance in 2005 after the mass vaccination campaign in 2004. However, a re-emergence of measles cases occurred in 2007, which was caused by other genotypes and the previous circulating genotype had disappeared. J. Med. Virol. 83:1424–1427, 2011. © 2011 Wiley-Liss, Inc.

## INTRODUCTION

In 2005, the Western Pacific Regional Office of the World Health Organization (WHO) set a goal of eliminating measles by 2012 [WHO, 2007]. In the Philippines, the Department of Health set a target to eliminate measles by 2008 [Department of Health, 2006]. Nationwide mass measles vaccination campaigns were conducted in 1998 (coverage: 94%), 2004 (95%), and 2007 (95%). The national measles surveillance in the Philippines monitors measles by using a sentinel surveillance system, which includes major hospitals in each region [National Epidemiology Center, 2004]. To define the proportion of actual measles cases among suspected cases, selected serum samples from suspected cases are sent to the Research Institute for Tropical Medicine in Manila for laboratory confirmation, which consists of detecting measles IgM antibody [Sobel et al., 2009]. Genotypes of circulating measles viruses (the order Mononegavirales, the family Paramyxoviridae, the genus *Mobillivirus*, Measles virus) can provide useful information on the source of infection and transmission pathway. However, such data are limited for the Philippines. This study describes the molecular epidemiology of measles viruses in the Philippines using stored serum samples.

## MATERIALS AND METHODS

Thirty-five serum samples positive for measles IgM antibody were selected at random for each year from 2000 to 2004. In subsequent years, all IgM-positive samples with sufficient volume were included. Viral RNA was extracted using the PureLink Viral RNA/DNA MiniKit (Invitrogen, Carlsbad, CA) and subjected to reverse transcription (RT). Standard PCR and nested PCR protocols were used to amplify the C-terminal hypervariable region of the N gene (456 nt) [National Institute of Infectious Disease, 2002]. PCR products were sequenced, and phylogenetic analysis was conducted using MEGA 3.1 [Kumar et al., 2004] by including reference strains for each genotype [WHO, 1999] and other D3, D9, and G3 sequences that are available in GenBank. Sequences described in this study were submitted to GenBank (AB514002-514030).

## RESULTS

Table I shows the measles surveillance data from 2000 to 2008. In 2004, the number of measles IgM-positive cases began to decline, and no positive samples were detected in 2005. However, in 2007, an increase in the number of cases was detected. From 7,836 laboratory confirmed cases, 409 samples were tested for the measles gene by RT-PCR. In total, 30 samples were positive for the N gene (Table I).

**Table I:** The Number of PCR Positive Cases Based on N Gene Among Sample Tested for PCR and the Results

Year	Samples tested for ELISA	Measles IgM positive	Samples tested for PCR	Sample positive for PCR (N)	Genotypes detected
2000	2,506	2,193	35	9	D3
2001	2,665	2,143	35	6	D3
2002	2,552	2,124	35	5	D3
2003	1,225	1,081	35	4	D3
2004	221	155	35	1	D3
2005	71	0	Not tested	Not tested	Not tested
2006	114		3	0	
2007	569	174	64	4	D9.G3
2008*	523	167	122	1	D9
Total	10,446	7,836	364	30	

* AsofM.25th200S, RITM.

**Fig. 1:**
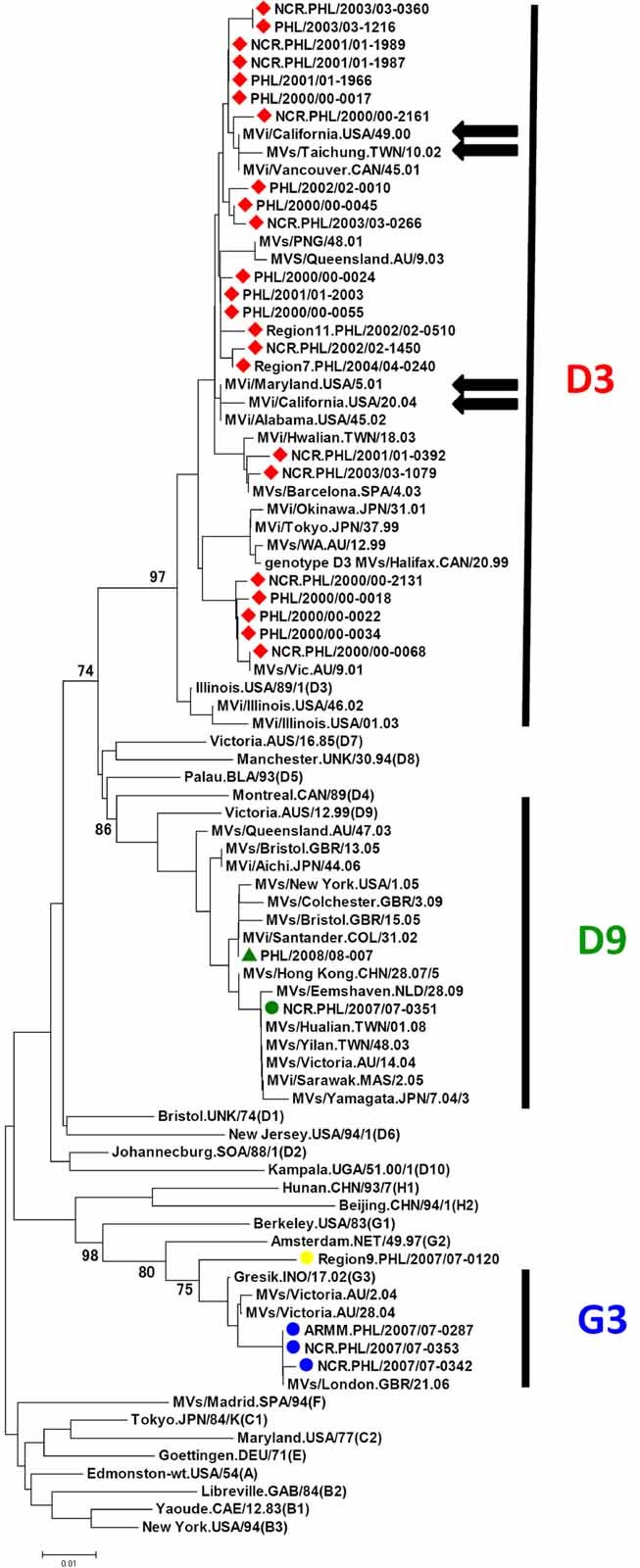
Genotyping of measles virus strains from the Philippines by sequencing and phylogenetic analysis based on the 450 C-terminal nucleotides of the N gene by using the Neighbour-Joining method. A square indicates the strains that were isolated until 2004, a circle indicates the strains collected in 2007 and a triangle indicates the strains collected in 2008. Genotypes are indicated by color as follows: Red: D3, Green: D9, Yellow: unclassified and Blue: G3. The arrow indicates the sequence of imported cases from the Philippines. The percentage of replicate trees in which the associated taxa clustered together in the bootstrap test (1,000 replicates) is shown next to the branches.

**Fig. 2:**
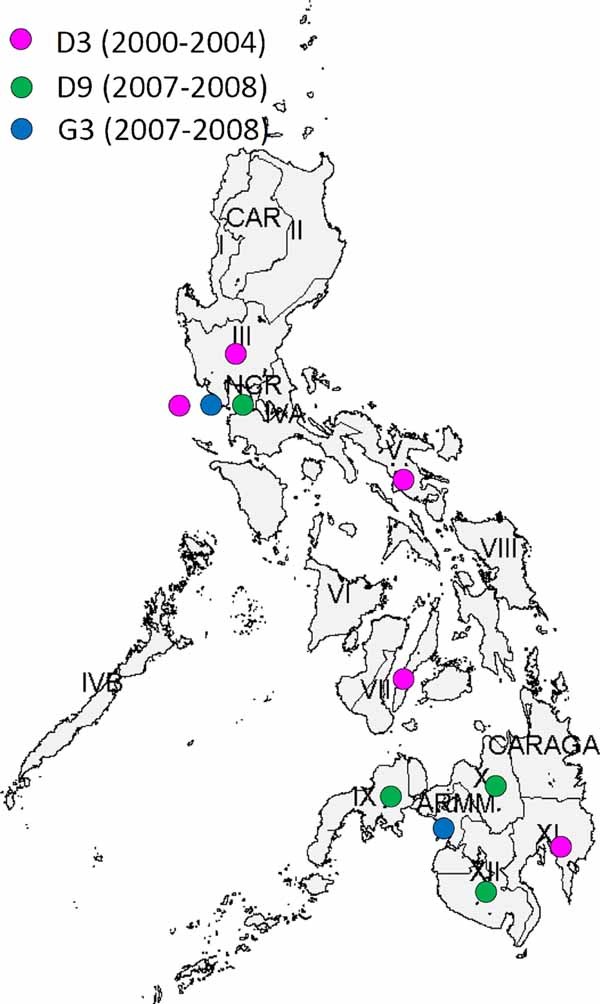
Geographical distribution of detected measles genotypes in the Philippines between 2000 and 2008. Region name and number are shown. CAR, NCR, and ARMM indicate Cordillera Administrative Region, National Capital Region, and Autonomous Region in Muslim Mindanao, respectively.

All samples collected between 2000 and 2004 from five regions [National Capital Region (NCR), Regions 3, 5, 7, and 11] were classified as genotype D3 (Fig. 1). However, samples collected in 2007 and 2008 were classified as D9 and G3 (Fig. 1). D9 was found in two regions (NCR and Region 9) (Fig. 2). G3 was also found in two regions [NCR and Autonomous Region in Muslim Mindanao (ARMM); Fig. 2]. Both D9 and G3 were detected in NCR, where only D3 had been detected previously.

One sample (Region9.PHL/2007/07-0120) was not classified as any of the known genotypes (Fig. 1) (AB514025). The sequence differences between Amsterdam.NET/49.97(G2) and Region9.PHL/2007/07-0120 were 4.4% and 4.9% in the N and H genes (partial H gene), respectively. The sequence differences between Gresik.INO/17.02(G3) and Region9.PHL/2007/07-0120 were 2.7% and 2.7% in the N and H genes (partial H gene), respectively. Sequencing of the whole H gene for Region9.PHL/2007/07-0120 was not possible due to insufficient viral RNA content in the sample.

## DISCUSSION

This study demonstrated that the circulating genotype in the Philippines was D3 until 2004. This result confirmed previous findings that the genotype of imported cases with epidemiological links to the Philippines was D3 [Rota et al., 2002, 2004; WHO, 2005a; Cheng et al., 2009]. All of those importations of D3 from the Philippines were reported between 1989 and 2002. The sequences of D3 detected in the Philippines were placed within at least two lineages and did not cluster with the D3 reported from Japan and Papua New Guinea (Fig. 1). The genotype change in 2007 occurred simultaneously with the increase in the number of measles cases. Before this change, the nationwide mass measles immunization campaign “Ligtas Tigdas” that targeted all children aged 9–48 months was conducted in February 2004 [Department of Health, 2006]. The data suggested that the vaccination campaign in 2004 covered a sufficient proportion of the target age group and succeeded in interrupting the chain of transmission of the endemic measles strain [Sobel et al., 2009]. After D3 disappeared in the Philippines, D3 had not been reported [WHO, 2008]. The documented importation cases from the Philippines in 2009 and 2010 were also D9 [WHO, 2010]. Those facts support the finding that D3 circulation in the Philippines, which used to be a possible major source of D3 has ended. The reported incidence of measles per 100,000 population was less than one based on the sentinel surveillance system in 2005 [WHO, 2005b, 2006]. However, the number of measles cases started to increase again in 2007. This resurgence may have been caused by newly introduced genotypes. Similar shifts in genotype have been reported in other countries such as Spain [Rima et al., 1997] and Germany [Santibanez et al., 2002]. Changes in circulating measles genotypes can occur in countries that have suboptimal control programs. In such situation, interruption of transmission for a short period may be possible, but failure to maintain sufficient herd immunity can result in the resurgence of measles due to introductions of the virus that spreads quickly among the susceptible population that has been accumulated gradually. A second follow-up measles immunization campaign in the Philippines was conducted between 15th October and 15th November, 2007 [Department of Health, 2006]. However, the number of laboratory confirmed cases continued to increase in 2008 (Fig. 1). Because of the high infectivity, 93–95% herd immunity is required to interrupt measles transmission [Moss, 2007]. A sustainable vaccination strategy that includes both routine immunization and supplemental immunization activity is needed to achieve such a high immunity level. The D9 and G3 genotypes have been circulating mainly in the Asia-Pacific Region. According to recent reports, D9 was detected in Indonesia, Hong Kong, Singapore, and New Zealand, and G3 was detected in Indonesia, Australia, and New Zealand [WHO, 2007, 2008]. Among D9 variants, one strain (NCR.PHL/2007/07-0351) exhibited 100% homology with measles genotypes detected in Taiwan (Yilan.TWN/48.03, Hualian.YWN/01.08), Australia (Victoria.AU/14.04), and Malaysia (Sarawak.MAS/2.05). However, the other strain (PHL/2008/08-007) was identical to a strain from Colombia (Santander.COL/31.02; Fig. 2). This suggests that the importation event might have occurred more than once. G3 detected from the Philippines showed 100% homology with the genotype from England (Mvs/London.GBR/21.06). G3 strains detected in NCR (NCR.PHL/2007/07-0353) and ARMM (ARMM.PHL/2007/07-0287) were also 100% homologous despite a long distance between the two regions (Fig. 2). This may indicate that G3 was imported into the Philippines once and subsequently spread throughout the country. The route of importation to the Philippines could not be identified because of the lack of detailed epidemiological information and incomplete genetic information for other countries. To eliminate measles worldwide, efforts in a single country are insufficient due to frequent cross-border transmissions.

The current study has several limitations. First, only serum samples were available, and the PCR-positive rate was low due to the low viral RNA content in serum and the timing of sample collection [Riddell et al., 2001]. Second, one strain could not be classified as any of the existing genotypes. The sequence of this strain is located between G2 and G3. However, it could not be identified as G2 or G3. According to the past proposal for new genotypes, new genotypes are designated if the nucleotide sequence differs from the closest reference sequence by at least more than 2.5% in hypervariable region of N and 2.0% in full length H gene [WHO, 2001]. The N gene nucleotide sequence of this strain differs enough to be classified as new genotype, but it was not possible to amplify the full H gene sequence. In addition, the detection of such unidentified strain was limited to one sample. The collection of nasopharyngeal swabs or leukocytes for isolating the virus is necessary in order to characterize fully the genotypes of circulating measles viruses.
